# A New Method of Making Palatable and Digestive Milk

**Published:** 1895-07

**Authors:** 


### A NEW METHOD OF MAKING PALATABLE AND DIGESTIVE MILK.

Dr. Robert T. Edes, of Boston, gives a valuable way of preparing milk where other methods have not proved useful:

A pint of milk is gently warmed. Into it is dropped, very slowly and with constant stirring, about twenty minims of the dilute hydrochloric acid of the United States Pharmacopœia. The milk should be stirred until it cools. In this way a very fine flocculent coagulum is produced, floating in the whey, which is easily accessible to the digestive secretions, while the whole fluid has lost somewhat of the flat and cloying taste which makes it unacceptable to so many. It will be noticed that milk prepared in this way differs from the various "wheys" in the highly important particular that the casein is retained and used, instead of being separated out as a distinct product, while it avoids the bitterness of pancreatinized milk.—*Boston Medical and Surgical Reporter.*

